# Population Characteristics of Cats Adopted from an Urban Cat Shelter and the Influence of Physical Traits and Reason for Surrender on Length of Stay

**DOI:** 10.3390/ani9110940

**Published:** 2019-11-08

**Authors:** Hannah Miller, Michael Ward, Julia A. Beatty

**Affiliations:** Sydney School of Veterinary Science, Faculty of Science, University of Sydney, NSW 2006, Australia; hmil4670@uni.sydney.edu.au (H.M.); michael.ward@sydney.edu.au (M.W.)

**Keywords:** welfare, adoption, feline, shelter-housed, coat color, white, stray, cat, length of stay, rehome

## Abstract

**Simple Summary:**

Every year, millions of cats are admitted to shelters around the world. Reducing the amount of time that cats stay in a shelter environment (length of stay, LOS) promotes animal welfare by reducing illness and stress, as well as supporting more efficient use of shelter resources. Understanding the factors that might influence LOS supports evidence-based interventions aimed at improving the flow of animals through shelters. Whether the same factors affect LOS in shelters of different types and from different geographic regions is poorly understood. We studied cats adopted from an urban shelter in Sydney, Australia, and found that stray cats have a longer LOS than owner-relinquished cats, supporting the results of previous studies. Surprisingly, in contrast to the widely held view that black cats stay in shelters longer than white cats, the opposite was true here—overall, white cats stayed longer than black cats in the shelter, even when other factors such as age were taken into consideration. Shelters might consider analyzing their own data, where possible, to inform strategies to reduce LOS.

**Abstract:**

Measures aimed at reducing the length of stay (LOS) of cats in shelters can promote animal welfare and more efficient use of resources. The extent to which variables shown to impact LOS are broadly applicable is unclear. The aim of this study was to describe a population of cats adopted from an urban shelter, and to analyze the association between potential predictor variables and LOS. A study cohort was identified retrospectively from shelter records (*n* = 2584), 48.8% of which were < 12 weeks old at admission, and 80.7% were stray. Among 445 cats relinquished by owners, reasons for surrender were primarily owner-related (87.2%). Overall, reason for surrender and coat color were significantly associated with LOS. Hazard ratios showed that all reasons for surrender for owner-relinquished cats were associated with a shorter LOS than stray cats and this association was significant (*p* < 0.05) for all except cat behavioral or medical reasons. In contrast to previous reports, white cats had a significantly (*p* < 0.05) longer LOS than black cats. This study highlights an important role for shelter-specific baseline data to inform and measure the effect of interventional studies aimed at improving animal welfare by reducing LOS in shelter-housed cats

## 1. Introduction

The relinquishment of cats to shelters is a universal phenomenon with diverse and complex drivers [1,2]. Recent data from Australia and the USA indicate declining euthanasia rates, although 24–40% of cats admitted to shelters are still euthanized for reasons often related—either directly or indirectly—to limited resources [3,4].

Shelters are a heterogeneous group of organizations operating in a range of communities. A resource-limited environment is common. Measures that reduce the length of stay (LOS) support the more efficient use of available resources and are associated with positive welfare benefits such as reducing the incidence of upper respiratory tract disease and negative behavioural outcomes [5,6,7]. This relationship is not universal—some measures, for example, euthanasia, reduce LOS but do not promote welfare. Investigating factors that influence LOS among cats that are ultimately adopted provides an evidence base for targeted interventions and policy changes that aim to reduce LOS to promote positive animal welfare outcomes.

Studies investigating the effect of variables—including age, breed, sex, coat color and pattern, origin (stray or owner-surrendered), environmental enrichment, and adopter perceptions—on LOS are increasing in number [3,8,9,10,11] While these studies provide very useful information, they have been conducted in shelter facilities that differ in size, population characteristics, euthanasia policies, and geographic region. As more studies become available, it will be important to identify those factors that might consistently influence LOS, so that they can be confidently applied to management of shelters broadly.

The aim of this study was to describe the population characteristics and LOS of cats admitted to an urban shelter in Sydney and to investigate the association between potential predictor variables and LOS.

## 2. Materials and Methods

### 2.1. Shelter Facility

Data were obtained from a shelter located in metropolitan Sydney, Australia. Admissions are accepted from members of the public (owner-surrendered and stray cats) as well as veterinary clinics and municipal shelters. Shelter intake is limited so that capacity is not exceeded. The shelter facilities and polices are consistent with the Capacity for Care (C4C) program to reduce stress, reduce illness, and facilitate the flow of cats and kittens through the shelter [12]. Cats are euthanized only on veterinary advice or if they test seropositive for feline immunodeficiency virus (FIV), testing being routine only for cats considered to be at risk. Data are collected routinely by shelter staff and entered into a cloud-based customer relationship management database (Salesforce, Melbourne VIC, Australia) for every cat admitted.

### 2.2. Case Selection

The database was searched for cases entering the shelter from 1st January 2016 until 20th March 2019 inclusive. Data were exported to Microsoft Excel. Cases were included if the following data were available; date of admission, date of rehoming, age at admission (known or estimated), sex, breed, coat color, and reason for surrender. Cases were excluded if they were not yet adopted, had been reunited with their owner, were euthanized, or if data for any variable was missing.

### 2.3. Data Categorization

Age on admission, provided by the owner or estimated by shelter staff, was categorized as <12 weeks, ≥12 weeks and ≤52 weeks, >52 weeks and ≤2 years, >2 years and ≤7 years, or >7 years and <10 years. The oldest recorded cat in the shelter during the study was 9.5 years old. Since all cats were de-sexed prior to becoming available for adoption, sex was categorized as male or female. To categorize breed, all mixed-breed cats (domestic shorthair, medium hair, and longhair) were combined, as were all purebred cats. Coat length was categorized as short-haired or long-haired (including medium-haired). A single, mutually-exclusive, primary coat colour category was assigned to each case; black, red (including ginger or champagne), tabby (including silver, classic, dark, ticked), tri-coloured (including tortoiseshell or calico), white, grey (including grey, charcoal, silver, blue), other (point colors, oriental or brown). The primary color was the predominant coat colour. The term stray was used to describe any case for which no owner was identified, comprising cats transferred from another facility, presented out of normal receiving hours or described as strays when presented by members of public. Reason for surrender was categorized as: stray, owner moving (to pet unfriendly accommodation or overseas), owner deceased, human medical reasons (allergies or new baby), too many pets/financial hardship, cat behavior or medical problems, unwanted litter, or other reasons, including relationship breakdown and becoming homeless.

### 2.4. Length of Stay

Length of stay (LOS) was defined as the number of days from the date of admission (day 0) to the date of adoption inclusive. LOS is longer than time to adoption because of variable holding periods prior to becoming available for adoption. Holding periods facilitate de-sexing (at 1 kg body weight for kittens, which is usually reached at 10–12 weeks of age), veterinary health checks, and treatment of any illnesses for all cats. Stray cats are subject to a mandatory 7-day holding period and microchipped cats to a mandatory 14-day holding period. LOS was used as the outcome measure, rather than time to adoption, because it reflects the use of shelter resources and allows for comparison between studies [3,8].

### 2.5. Statistical Analysis

Descriptive statistics for LOS and predictor variables were calculated. The association between LOS and each potential predictor variable was assessed using the Kaplan–Meier estimator, in which adoption was the event of interest. Associations between LOS and each predictor were tested using a log rank (Mantel Cox) test. Age was considered a potential confounder *a priori*, and those predictors associated with LOS at *p* < 0.2 were then included in a Cox regression model to describe the effect (hazard ratio) of these variables on LOS. The assumption of proportional hazards over time was checked graphically. All statistical analysis was undertaken in SPSS^®^ statistics v24 (IBM, Armonk, NY, USA).

## 3. Results

### 3.1. Descriptive Analysis

An initial search of the database identified 2839 cases admitted during the 115-week study period. Reasons for exclusion were: not yet adopted (*n* = 114), euthanized (*n* = 87; 40 on veterinary advice, 37 testing FIV seropositive), missing data point (*n* = 45), and reunited with the owner (*n* = 9). Therefore, a cohort of 2584 cases was available for study.

A summary of the descriptive data obtained for this cohort is presented in Table 1. Almost half the cases (48.8%) were < 12 weeks of age on admission. The majority of cases were identified as stray (80.7%). Reason for surrender could be analyzed for 445 owner-surrendered cases (excluding “other” category). Most relinquishments related to owners perceiving too many animals, financial reasons, moving or human medical reasons (87.2%). Cat-related behavior or medical condition, or a deceased owner, were cited in a minority of surrenders (6.1% and 6.7% respectively).

### 3.2. Influence of Variables on Length of Stay

**Figure 1:**
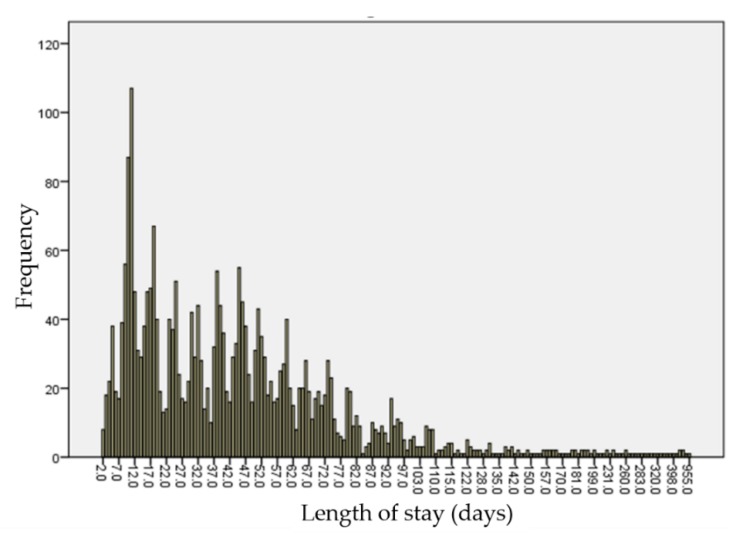
Frequency distribution of length of stay for 2584 cats adopted from an urban shelter.

Length of stay was non-normally distributed (right-skewed; Wilks–Shapiro test, W = 0.616, *p* < 0.001) (Figure 1). The median LOS for the entire cohort was 39 days, (range 2–955 days, IQR 17–60 days). On univariate analysis, age at admission (*p* < 0.01), coat color (*p* < 0.01) and reason for surrender (*p* = 0.01) were significantly associated with LOS (Table 1). No significant preference for rehoming males over females (*p* = 0.16), purebred over mixed breed (*p* = 0.76), or shorthaired over longhaired cats (*p* = 0.32) was identified.

Adjusting for age (as an *a priori* potential confounder), coat colour (*p* = 0.01) and reason for surrender (*p* < 0.01) remained significantly associated with LOS. In multivariate analysis, both coat colour and reason remained significantly (*p* < 0.01) associated with LOS, adjusting for age. In this final model (Table 2), the only significant (*p* < 0.05) pairwise difference identified for coat color was white versus black; white cats had a significantly longer LOS (a lower hazard ratio) than black cats. All reasons for surrender for owner-relinquished cats were associated with a shorter LOS (a higher hazard ratio) compared to stray cats and this association was significant (*p* < 0.05) for all except cat behavioral or medical reasons (*p* = 0.17). The interaction between coat color and reason was not statistically significant, *p* = 0.58.

## 4. Discussion

The structure of the cohort investigated here, comprising predominantly young cats, most with no identifiable owner, is common among descriptions of shelter populations studied previously [13,14,15,16,17]. Regarding owner-relinquished cats, our finding that cat-related reasons for surrender were uncommonly cited, in comparison to human-related reasons, is in line with the findings of recent Australian studies where accommodation-related, personal, and financial reasons were commonly provided by owners surrendering cats [1,13,18]. Together, these studies support the increased acceptance of pets in rented accommodation in Australia.

It is expected that LOS for kittens may be longer than adults because of the requirement to hold kittens until they reach 1 kg in body weight, when they can be de-sexed. This effect on LOS has been reported previously [3]. However, as age increased further, LOS decreased, so that cats over 2 years of age were adopted 23% quicker, and cats aged between 7 and 10 years over 53% quicker than kittens. Geriatric cats (over 10 years) are not included here, as none were identified during the study period. In contrast, a study at a no-kill shelter in the USA, that calculated LOS similarly, found the inverse, i.e., that LOS increased as the cats’ age increased [8]. The reasons for this difference cannot be ascertained, but it demonstrates that caution should be exercised before extrapolating findings between studies.

Several studies conducted outside Australia show that black coat color is associated with a longer or the longest LOS [3,8,19]. In contrast, in this shelter, white cats had the longest LOS, significantly longer than black cats in the final model, demonstrating that the relationship between coat color and LOS can vary between geographic regions. The reasons underlying the observed association between coat color and LOS are not investigated here. Coat color has been identified by potential adopters as an important selection criterion [20]. It can be speculated that adopter preference is one potential influence LOS in cats of different primary coat colors. A previous study from Australia identified that when cats were grouped by color, black cats had the highest proportion of cats adopted, although the small sample size precluded statistical evaluation of the influence of coat colour on adoption [21]. It is worth noting that advice to keep white cats strictly indoors to reduce the risk of cutaneous squamous cell carcinoma is common in Australia. In this shelter, advice and factsheets on keeping cats indoors were given to potential adopters of cats for a range of reasons, including white ears or nose in cats of any primary color. The effect of specific advice on a range of issues could be investigated in a future controlled study.

In the final model, cats surrendered with no identifiable owner were housed in the shelter longer than owner-relinquished cats, regardless of the reason given by owners for surrender. It has been suggested that a bias against adopting stray cats may exist, based on the assessment of cats’ interactions with adopters and adopters’ perceptions of cats described as strays [9]. Whether such a bias might be one variable influencing LOS in this population could be assessed indirectly in the future by determining the effect of strategies to promote the adoption of stray cats on LOS, using these data as a baseline.

There are several limitations to our study. Holding periods contribute to LOS and these do not necessarily affect all admissions equally, as noted previously for kittens awaiting de-sexing. The retrospective nature of the study means that further details of each variable are not available, for example, a single reason for owner relinquishment was recorded here. This almost certainly oversimplifies what has been identified as a multifaceted, complex phenomenon in studies using survey responses to analyze relinquishment decisions [1]. It should also be remembered that information given by owners may be, knowingly or unknowingly, misreported and these data are largely unverifiable. Another limitation is that not all variables that might influence LOS are expected to be not recorded in the database. Future studies might consider including, for example, health status or interactions between potential adopters and cats available for adoption, which has been shown to influence LOS [21]. Finally, the small sample size of some categories likely led to insufficient study power to detect differences, for example, for breed where relatively few purebred cats were included.

Identifying variables that are associated with LOS does not imply a causal association. However, it can inform the design of interventions for which influence on LOS can then be quantified. Interventions that result in reduced LOS can then be considered for adoption as shelter policy. In the shelter studied here, the effects of interventions to promote the adoption of white cats and cats that are relinquished with no identifiable owner could be tested to determine any quantitative effects on LOS.

## 5. Conclusions

Given the variability in findings between published reports and the increasing use of computerized record-keeping systems, shelters may elect, where possible, to analyze their own data to design and monitor the effectiveness of interventions to reduce LOS.

## Figures and Tables

**Table 1:** Descriptive summary for 2584 cats adopted from an urban shelter and variables significantly associated with length of stay (LOS) on univariate analysis.

Variable	Category	Number of Cases (% of Total) ^a^	Median LOS (Days)	Range-Lower	Range-Upper	IQR
Age	< 12 weeks	1262 (48.8)	39	2	561	42
	≥ 12 weeks ≤ 52 weeks	728 (28.2)	40	2	955	46
	> 52 weeks ≤ 2 years	394 (15.2)	37	2	383	42
	> 2 years ≤ 7 years	148 (5.7)	30	3	181	41
	> 7 years < 10 years	52 (2.0)	18	5	199	30
Sex	Male	1300 (50.3)	NS	NS	NS	NS
	Female	1284 (49.7)				
Breed	Mixed breed	2561 (99.1)	NS	NS	NS	NS
	Purebred	23 (0.9)				
Primary colour	Black	648 (25.1)	38	2	561	43
	Red	411 (15.9)	36	2	298	41
	Tabby	958 (37.1)	38	2	331	42
	Tri-colored	255 (9.9)	43	2	398	44
	White	144 (5.6)	45	4	955	57
	Grey	142 (5.5)	44	3	179	37
	Other	26 (1.0)	38	9	192	54
Reason for surrender	Stray	2084 ^b^	43	2	955	46
	Owner moving	78 (17.5)	19	5	181	31
	Owner deceased	30 (6.7)	18	7	82	26
	Human medical reason	99 (22.2)	34	6	131	29
	Too many pets/financial	119 (26.7)	35	6	101	36
	Cat behavior or medical	27 (6.1)	31	5	83	48
	Unwanted litter	92 (21.7)	18	5	88	26
	Other	55 ^b^	11	4	39	8

^a^ may not total 100% because of rounding; ^b^ Stray and Other categories excluded from calculation of % of owner-relinquishments with reason recorded (*n* = 445); IQR = interquartile range; NS = non-significant.

**Table 2:** Risk factors identified for adoption of cats from an urban shelter, adjusted for age. Hazard ratios represent the “risk” of adoption and a hazard ratio > 1 indicates a shorter length of stay.

Variable	Category	Coefficient (B)	SE	*p*-Value	Hazard Ratio	95% CI
Reason for surrender	Stray	0	–	–	1.0	
	Owner moving	0.58	0.13	<0.01	1.78	1.39–2.28
	Owner deceased	0.98	0.2	<0.01	2.67	1.81–3.93
	Human medical reason	0.26	0.11	0.02	1.30	1.04–1.62
	Too many pets/financial	0.38	0.1	<0.01	1.47	1.22–1.77
	Cat behavior or medical	0.28	0.2	0.17	1.32	0.89–1.95
	Unwanted litter	0.78	0.11	<0.01	2.19	1.77–2.70
	Other	1.62	0.14	<0.01	5.05	3.81–6.69
Colour	Black	0	–	–	1.0	
	Red	0.10	0.06	0.11	1.11	0.98–1.26
	Tabby	0.07	0.05	0.16	1.07	0.97–1.19
	Tri-coloured	−0.05	0.07	0.5	0.95	0.82–1.10
	White	−0.33	0.09	<0.01	0.72	0.6–0.86
	Grey	0.1	0.09	0.31	1.10	0.92–1.32
	Other	−0.16	0.20	0.43	0.86	0.58–1.27
